# Statistical methodology for constructing gestational age‐related charts using cross‐sectional and longitudinal data: The INTERGROWTH‐21^st^ project as a case study

**DOI:** 10.1002/sim.8018

**Published:** 2018-11-28

**Authors:** Eric O. Ohuma, Douglas G. Altman, for the International Fetal and Newborn Growth Consortium for the 21st Century (INTERGROWTH‐21st Project)

**Affiliations:** ^1^ Nuffield Department of Women's & Reproductive Health University of Oxford, John Radcliffe Hospital Headington, Oxford, OX3 9DU UK; ^2^ Centre for Statistics in Medicine, Botnar Research Centre, Nuffield Department of Orthopaedics, Rheumatology and Musculoskeletal Sciences University of Oxford Windmill Road, Oxford, OX3 7LD UK; ^3^ Centre for Tropical Medicine and Global Health, Nuffield Department of Medicine University of Oxford Old Road Campus, Oxford OX3 7BN UK

**Keywords:** cross‐sectional, human growth, longitudinal, statistical methodology

## Abstract

Most studies aiming to construct reference or standard charts use a cross‐sectional design, collecting one measurement per participant. Reference or standard charts can also be constructed using a longitudinal design, collecting multiple measurements per participant. The choice of appropriate statistical methodology is important as inaccurate centiles resulting from inferior methods can lead to incorrect judgements about fetal or newborn size, resulting in suboptimal clinical care.

Reference or standard centiles should ideally provide the best fit to the data, change smoothly with age (eg, gestational age), use as simple a statistical model as possible without compromising model fit, and allow the computation of Z‐scores from centiles to simplify assessment of individuals and enable comparison with different populations. Significance testing and goodness‐of‐fit statistics are usually used to discriminate between models. However, these methods tend not to be useful when examining large data sets as very small differences are statistically significant even if the models are indistinguishable on actual centile plots. Choosing the best model from amongst many is therefore not trivial. Model choice should not be based on statistical considerations (or tests) alone as sometimes the best model may not necessarily offer the best fit to the raw data across gestational age. In this paper, we describe the most commonly applied methodologies available for the construction of age‐specific reference or standard centiles for cross‐sectional and longitudinal data: Fractional polynomial regression, LMS, LMST, LMSP, and multilevel regression methods. For illustration, we used data from the INTERGROWTH‐21^st^ Project, ie, newborn weight (cross‐sectional) and fetal head circumference (longitudinal) data as examples.

## 1 INTRODUCTION

A reference or standard chart depicts a family of curves representing a few selected centiles of the distribution of some physical characteristic of the reference population as a function of age. Growth charts aid clinical judgements and are primarily used: to compare attained size with reference data when gestational age (GA) is known at a specified time,^1^ to estimate GA from attained size (eg, crown‐rump length, fetal head circumference (FHC), etc),^2^, ^3^, ^4^ and to assess a fetus's growth between two time points (velocity).^5^, ^6^

The choice of analysis is usually informed by study design. Reference or standard charts, eg, fetal and newborn charts, are mostly based on cross‐sectional data because single‐visit data is easiest to collect^7^; each fetus or newborn is measured only once. A variety of statistical methods for constructing reference or standard charts has been suggested, including parametric, semiparametric, and nonparametric techniques. Detailed overviews and comparisons of different approaches can be found in the literature.^8^, ^9^, ^10^, ^11^, ^12^ However, longitudinal data are becoming more common and analysis of repeated measures data pose analytical challenges that require different analysis techniques to single measure data, because such data deviate from the independence of observations assumption that most classical statistical methods are based on.

Growth charts are used as a screening tool in the identification of fetuses or newborns as small, appropriate, or large for a specified GA, based on specified centiles on a reference or standard chart.^13^, ^14^ Early detection of fetal growth restriction or macrosomia may decrease associated morbidity and mortality whereas accurate information on GA may prevent unnecessary obstetric interventions at the time of delivery. For example, a fetus classified as being >97^th^ centile according to an estimated fetal weight chart would help clinicians in making judgements as to whether to deliver early or consider a caesarean section to avoid complications that are associated with delivering a large baby. Values outside extreme centiles are indicative of growth restriction, excessive growth, or other clinical complications affecting growth.

The aim of this paper is to give a brief overview of common methodology used for deriving reference or standard charts of attained size based on a cross‐sectional design and an extension of methodology for longitudinal data. We demonstrate and compare statistical methodologies for constructing GA‐related size charts from cross‐sectional data using fractional polynomial (FP) regression,^15^ LMS,^11^ LMST, LMSP methods,^16^, ^17^ and multilevel regression methods for longitudinal data. Multilevel models that account for data dependency with varying complexity are used. These approaches are compared by evaluating model fit using goodness‐of‐fit statistics and diagnostic plots.

The statistical methods adopted to create centile curves as a function of age should fulfil certain criteria, ie, (i) the centiles should change smoothly with age; (ii) the fitted curves should be a good fit to the data, especially the outer centiles (eg, the 3^rd^ and 97^th^ centiles) where uncertainty is greatest and the regions between and outside the centile curves should contain the appropriate fractions of the reference sample at all ages, eg, the proportion of data below the 3^rd^ and 97^th^ centiles is expected to be 3%; (iii) centiles should never cross; (iv) ideally Z‐scores and centiles can be obtained simply for future individuals; (v) apply continuous age smoothing, not age binning; (vi) for nonnormal data at each age, there should be flexibility to account for skewness and kurtosis; and (vii) the models should be as simple as possible consistent with the aforementioned requirements. The principle of smooth centiles according to GA relies on the underlying distribution moments being smooth. For example, the LMS method^11^ achieves this by assuming that the measurement at each GA can be transformed to a normal distribution using a Box‐Cox transformation, and just three parameters (the Box‐Cox power λ, the median μ, and the coefficient of variation σ) to summarise the distribution. The three quantities are allowed to change smoothly with age, reflecting the changing underlying distribution. In addition, modelling approaches should be able to account for increasing variability with GA, which is a phenomenon usually observed in growth data.^15^

In this paper, we describe and compare some, but not all, existing statistical methods that can achieve the aforementioned goals. Issues dealing with the assessment and impact of ignoring data dependency for longitudinal data are not discussed as they are beyond the scope of this paper.

## 2 METHODOLOGY BACKGROUND

### 2.1 A parametric approach

A parametric approach relies on making inferences from a known distribution and captures all its information about the data within its parameters. These approaches are based on certain assumptions about the distribution of the data, eg, normality of observations or transformation of the observations to a normal distribution. By making reference to a certain distribution, say the normal, we rely on parameters that define that distribution, eg, the mean and standard deviation. A normality assumption is the basis of most statistical methods and is thus commonly applied in data analysis. In the current context, the issue is whether the measurements of fetuses or newborns are normally distributed at a specific GA.

When the normality assumption is violated, logarithmic transformation is commonly used due to its desirable mathematical properties of back‐transformation to original values,^18^ ease of fit, and variance stabilisation.^19^ Logarithmic transformation can be extended to the shifted logarithmic transformation of the form log ( *y* + *k*). This involves simply adding or subtracting a constant, *k*, to all observations, its sign related to whether the distribution of the dependent variable is negatively or positively skewed. Although it is rarely used in practise, a good example was in the National Study of Growth and Health by Rona and Altman where they modelled weight as log (weight – constant (*k*)) for boys and girls separately, and *k* varied by age.^20^ To obtain estimates in the original scale, the final model is first back‐transformed using antilog, then the constant, *k*, is subtracted. The more recent generalised additive modelling of location, scale, and shape technique (GAMLSS) offers a wide variety of distributional forms, of which the normal distribution is the simplest with just two parameters, location (mean, μ) and scale (SD, σ).

New approaches for fetal and neonatal size reference construction extend these two‐parameter models to three‐ and four‐parameter models by exploring more flexible distributions that may offer a better representation of the data. Of note is the following: the quantitative element of model comparison relates to the ubiquitous trade‐off between parsimony and goodness‐of‐fit. Whilst highly complex models may offer the best fit to the data, an objective assessment of the gain in model fit versus model complexity ought to be considered also. The importance of parsimony is key to good scientific principles and one such famous principle by Ptolemy is widely known as Occam's razor.

Data that is not normally distributed can be modelled by complex models which in addition to modelling μ and σ, model shape parameters also, ie, ν, for measuring skewness, and τ, for measuring kurtosis. Both skewness and kurtosis mainly affect the most extreme tails of the distribution. Skewness rather than kurtosis has been shown to be common with growth data^21^, ^22^ and therefore should be considered during modelling. A common methodology that accounts for skewness but not kurtosis is the LMS method.^11^ Generalised additive modelling of location, scale, and shape is an extension of the LMS and has the advantage of adjusting for kurtosis as well as skewness.^23^ However, for growth data, kurtosis have little influence on fitted centiles as was demonstrated by the WHO in the construction of child growth standards.^24^

A justification for sometimes considering multiple models with the same complexity, ie, same number of parameters is because different distributions model either skewness or both skewness and kurtosis. For example, the power exponential distribution^25^ is a three‐parameter distribution (μ, σ, and τ), and is suitable for data with higher kurtosis (leptokurtic) and lower kurtosis (platykurtic) than the normal distribution. The four parameter Box‐Cox t‐distribution is best for leptokurtic data whereas the Box‐Cox Power Exponential distribution models both leptokurtic and platykurtic data. Further details of the various distributions, their respective number of parameters, and whether they are suitable for modelling skewness (positive or negative) or kurtosis (leptokurtosis or platykurtosis) or both are presented in the GAMLSS instructions manual.^26^

### 2.2 A nonparametric approach

A nonparametric approach does not make any distributional assumptions about the data and thus can capture more subtle aspects of the data. Inevitably, situations may arise when data are nonnormal even after transformation. In such situations, it would be desirable to consider alternative methods of estimating reference curves that impose less stringent global hypotheses on the form of the conditional distributions. One such approach is the use of quantile regression for reference models, as described by Wei et al,^12^ based on the work by Koenker and Bassett.^27^ Advances brought by computer power have made it possible to estimate the distributions directly by estimating their quantiles. Quantile regression is now a well‐established technique, and statistical software is available to fit quantile regression models.^28^ There are many applications of quantile regression techniques for modelling growth data and these have been shown to perform equally well as parametric methods.^12^, ^29^, ^30^, ^31^, ^32^ These methods allow quantiles to be estimated as a smooth function of age without making any distributional assumptions. Nonparametric methods lack a simple closed formula that can be easily written down and therefore limits its use clinically. For this reason, we did not consider nonparametric models and we do not discuss them further.

### 2.3 Statistical methods

Parametric approaches are common and preferred because of their properties and ease of understanding. Several methods are available for the construction of age‐related centiles, each with advantages and limitations.^33^ No single method is likely to be able to overcome all of the data modelling challenges associated with such data. Although some methods will be suitable for most situations, inevitably specific features unique to different methods will sometimes be desirable.

For example, it is well established that, on average, attained fetal size increase monotonically during pregnancy. The between‐subject variability of fetal dimensions also tends to increase with GA. It is therefore crucially important to consider both the relation between the average attained fetal size and GA, and how variability (SD) changes with GA.^10^ The FP regression approach offer nonlinear functions of GA for most fetal dimensions. Therefore, one of the most used method for construction of attained size charts fits separate models for the mean and SD using FPs. This approach assumes normality of fetal dimensions conditional on GA.^34^

In January 2003, the WHO convened a group of statisticians and child growth experts to review available methods for constructing age‐related centiles and develop a strategy for assessing their strengths and weaknesses.^35^ The group reviewed 30 methods for attained growth curves and agreed on four methods, ie, FP regression method,^34^ LMS (λ, μ, and σ) method,^11^, ^21^, ^35^ LMST^16^ method, and LMSP^17^ method. All four methods are parametric or semiparametric. In addition, we consider multilevel models for longitudinal data.

Based on the aims and considerations of the preferred modelling methods, coupled with findings from the WHO review and our systematic review of the methodological quality of studies designed to create fetal and neonatal anthropometric charts, we decided to focus on the most commonly used methods by summarising the underlying principles of each in turn. We further present the application of one method for analysing cross‐sectional data and another for longitudinal data using the INTERGROWTH‐21^st^ newborn weight and FHC data to illustrate and compare the methods.^7^, ^8^, ^9^, ^10^, ^15^, ^33^, ^36^

### 2.4 Analytical approaches for cross‐sectional data

#### 2.4.1 Fractional polynomial regression

Fractional polynomial regression is one of the most common parametric approach for modelling growth data especially during the prenatal period.^15^ It is based on the assumption that at each GA the measurement of interest has a normal distribution and that the mean and SD vary smoothly with GA. The C_α_ centile curve is estimated using

$$C_{100\alpha }\left(t\right)=\mu \left(t\right)+Z_{\alpha }\times \sigma \left(t\right),$$

where C_100α_ (t) is the expected value of a given centile 100_α_ of the biometric trait at a given GA, Z_α_ is the normal equivalent deviate of size α (SD score or z‐score) corresponding to a particular centile, eg, Z_α_ = 1.88 for the 97th centile and −1.88 for the 3^rd^ centile, and μ(*t*) and σ(*t*) are the age‐specific mean and standard deviation, respectively, at the required GA for the reference population.

Originally, this approach involved several steps, ie, grouping age into groups (bins of age), which were then regressed on the mean age in each group using conventional polynomials of the form Y = β_0_ + β_1_*X_1_^p^_1_ + β_2_*X_2_^p^_2_ + β_3_*X_3_^p^_3_ + ..., where the exponents of X are nonnegative integers. Royston and Wright^37^ proposed starting with a cubic polynomial, and then reducing the number of terms sequentially if the regression coefficient of the highest term is not significantly different from zero. Once a suitable mean model is selected, a separate analysis is performed to model the variability around the mean. If the variable has a normal distribution at all ages, then the residuals should have a normal distribution and the absolute values of residuals should have the half normal distribution. In 1993, Altman proposed the regression of absolute residuals on length of gestation as a continuous variable hence avoiding the need to create GA groups. The age specific estimated SD can then be obtained by multiplying the fitted values by $\sqrt{\frac{\pi }{2}}\;$ as the values of absolute residuals should follow a half normal distribution.^38^ He further attempted to reweight the analysis for the mean using the fitted SD and this showed to only have little effect.

The FP regression method is based on least squares regression analysis, modelling the mean and SD centile curves as separate polynomial functions of GA. The FP regression method fits separate models for the mean and SD to account for the increasing variability with GA that is typical of fetal and newborn data. The method requires the assumption of a normal distribution.

Although conventional polynomials are popular, for growth data where variability increases with age, they suffer from many deficiencies. They offer only a few model shapes (low order polynomials), which often do not fit the data well, especially near the ends of the data range (high‐order polynomials). In addition, polynomial functions do not have asymptotes, so they can't model this type of behaviour. Royston and Altman^34^ and Royston and Sauerbrei^38^ introduced a generalisation of the polynomial function known as FPs, which is an extended family of curves.^34^ Fractional polynomials have the advantage of (i) parsimony, ie, they offer similar model fits as conventional polynomials but with fewer terms; (ii) flexibility as they provide a wide range of curve shapes, and (iii) the ability to approximate asymptotes.^39^

The linear predictor for a FP of order *m* for covariate X, denoted as FP*m (X)* with power terms p = ( *p*_1_ ≤ … ≤ *p*_m_), is given by FP*m* (X) = β_0_ + β_1_*X^p^_1_ + β_2_*X^p^_2_ + … + β_m_*X^p^_m_ where powers *p*_1,…,_ *p*_m_ are selected from a restricted set {−2,−1,−0.5,0,0.5,1,2,3} where *x* ^0^ denotes *log (x)* rather than x^0^ = 1. The degree of an FP model, *m*, is defined as the number of powers, *p,* of the explanatory variable, *X*. For example, a first‐order FP (FP1) with *p*_1_ = 0 will be of the form β_0_+ β_1_log(*X*). A second‐order FP (FP2) with *p*_1_ = −2 and *p*_2_ = 1 will be of the form β_0_+ β_1_ *X* ^*−*2^ + β_2_ *X*, and a third‐order FP, which also involves repeat powers, p = (0, 2*,* 2), will be given by β_0_ + β_1_log(*X*) + β_2_ *X* ^2^ + β_3_ *X* ^2^ log(*X*).

Fractional polynomials have been shown to perform well due to their great flexibility in allowing noninteger powers, logarithms, and repetition of powers,^34^, ^40^ a wide range of curve shapes, and because they have been shown to fit fetal data very well.^4^, ^10^, ^15^, ^34^, ^41^, ^42^, ^43^, ^44^ Usually, an FP of order m = 1 or m = 2 is sufficient for obtaining a good fit as they offer a wide variety of nonlinear (and linear) curves. The FP1 functions are always monotonic whereas FP2 regression models are either monotonic or nonmonotonic. Another advantage for FP2 over FP1 is the variety of models, ie, 32 possible models under FP2 compared to eight under FP1 regression models. For most applications, FP1 or FP2 regression models would suffice.^34^ The selection of the best FP powers can be obtained using an automated algorithm already implemented in the statistical software programmes STATA and R.

#### 2.4.2 LMS method

Van't Hof et al^45^ first suggested a method to deal with nonnormal anthropometry data. Using skewed skinfold data as an example, they suggested a power transform^46^ at each age to remove skewness, making the data approximately normally distributed. The proposed method consists of seven steps, which allows the power transform to change smoothly with age and to vary from one age to another. Cole^35^ generalised this method using three parameters λ, μ, and σ, the initials of which are, respectively, L, M, and S, giving rise to the name LMS method. The LMS method assumes that at each age the (biometric) measurement of interest follows a normal distribution after a Box‐Cox power transformation, λ. Therefore, data can be summarised by three age‐dependent functions λ, μ, and σ, such that the transformed outcome is a Z‐score with distribution close to N (0, 1). The location parameter, M(t), is the median rather than the mean (though for the normal distribution of course the two coincide), and S(t) represent the coefficient of variation (SD/median), of each biometric trait at each age. L(t) represents the value of the power needed to normalise the data at each age.

The three curves L(t), M(t), and S(t) are fitted using cubic splines by nonlinear regression and by maximising the penalised likelihood. Three smoothing parameters for the three curves are obtained (ie, equivalent (effective) degrees of freedom (edf)) from each fitted curve. The edf of each L(t), M(t), and S(t) curve is a measure of complexity and is interpreted as the dimensionality of the fitted function.^47^ For example, edf = 1 indicates a constant, edf = 2 refers to a straight line, edf = 3 is a quadratic curve, and edf ∼ 4 refers to more complex curve shapes. The choice of edf is somewhat subjective and is an indication of how well the data has been smoothed. It is desirable to strike a balance between model complexity (in terms of smoothing) and model fit to the raw data. The three curves together allow any centile to be calculated

$$C_{100\alpha }\left(t\right)=M\left(t\right)\;{\left(1+L\left(t\right)*S\left(t\right)*Z_{\alpha }\right)}^{\left(1/L\left(t\right)\right)},\;L\left(t\right)\neq 0\;$$

or

$$\;C_{100\alpha }\left(t\right)=M\left(t\right)\text{exp}\left(S\left(t\right)*Z_{\alpha }\right),\;L\left(t\right)=0,$$

where C_100α_ (t) is the expected value of a given centile 100_α_ of the biometric trait at a given age, M(t) is the median, S(t) is the coefficient of variation, L(t) is the power transform, and Z_α_ is the normal equivalent deviate of size α (SD score or z‐score). SD scores (SDSs) are recommended for making direct comparisons between different anthropometric measures and can also be used to compare different populations.^11^, ^35^

The SDS values for an individual can similarly be obtained

$$\text{SDS}={\left[y\left(t\right)/M\left(t\right)\right]}^{(L\left(t\right)}-1/L\left(t\right)*S\left(t\right),\;L\left(t\right)\neq 0\;$$

or

$$\;\text{SDS}=\text{log}\left[y\left(t\right)/M\left(t\right)\right]/S\left(t\right),\;L\left(t\right)=0,$$

where y(t) is the measured anthropometry at age *t*, M(t) is the median, S(t) is the coefficient of variation, and L(t) is the power transform at that age.^11^

#### 2.4.3 LMS extensions: the LMST and LMSP methods

The LMST method proposed by Rigby and Stasinopolous^16^ is an extension of the LMS method that models both skewness and kurtosis using the Box‐Cox t‐distribution. It can be used to model excess kurtosis over the normal distribution (leptokurtic data) when the Box‐Cox transformation fails to transform the data close to normality due to the presence of kurtosis. Similarly, the LMSP method proposed by Rigby and Stasinopolous^17^ is another extension of the LMS method that models both skewness and kurtosis. Unlike the LMST method, which can only model leptokurtic data, the LMSP method can model any type of kurtosis, ie, leptokurtosis, platykurtosis, or mesokurtosis. The LMSP achieves this greater utility by using the more flexible Box‐Cox power exponential distribution.

#### 2.4.4 Multilevel models

Multilevel linear models (also commonly referred to as hierarchical or mixed‐effect models) account for the dependence of observations by considering the hierarchical structure of the data and the correlation between measurements from the same fetus at different GAs, and also, if relevant, at the same GA for multiple measurements taken at each visit. They are regression equations that include both fixed and random components.^48^ The fixed components are the same for every subject and the random components differ between subjects. These methods allow each subject's growth pattern over time to be characterised. The between‐subject variability in the specified population can thus be quantified.

Multilevel models can vary in complexity and therefore account for the multilevel structure of the data in different ways. For example, the longitudinal design of the INTERGROWTH‐21^st^ FHC data resulted in a three‐level hierarchical data structure for FHC, ie, measurements within visits within participants. Level 1 is the triplicate measurements taken at each visit, FHC_1_, FHC_2_, and FHC_3_. Level 2 is the repeated ultrasound measurements taken for each woman over multiple visits during pregnancy. Level 3 is the measurements taken from many women in the eight recruitment sites (country).

In this paper, we therefore evaluated various multilevel models with increasing complexity, ie, a two‐level random intercept model, a two‐level random intercept and slope model, and a three‐level random intercept and slope model.

##### Model selection and diagnostics

Model comparison is as important as it is challenging. Here, we discuss several procedures for model comparison, with an emphasis on a trade‐off between model complexity (goodness of fit) and parsimony. The desire for centiles that are both smooth and precise is a tradeoff between the statistically best model and complexity of the model. Model choice should therefore not be based on statistical considerations alone.

The classical methods for selecting best models from regression analysis involve an assessment of the amount of variability in the data that is explained by the fitted model (R^2^ statistic). This approach has the limitation of not accounting for the modelling of the SD, which is crucial for growth data. Goodness of fit of the resultant models can be assessed by a plot of the residuals (observed values minus fitted values) according to age. A plot of the residuals against age shows how variability changes with increasing age. Formal statistical testing such the Akaike information criteria (AIC) and Bayesian information criteria (BIC), also called the Schwarz Bayesian criterion, can be considered when deciding whether to select a more complex model. The BIC penalises the deviance by log *n* units for each extra degrees of freedom (df), leading to optimal spline curves and a parsimonious final model. The AIC penalises the deviance by 2 units per extra df, which is a special case of the generalised AIC [k] or GAIC [k], where the penalty is *k* units of deviance per df.^49^ In addition, model fit can be visually evaluated using quantile‐quantile (Q‐Q) plots of the residuals, which can reveal any departures from normality; plots of residuals vs. fitted values; and the distribution of fitted Z‐scores against GA. Other approaches include a comparison of the estimated proportions of observations falling below a specified centile, eg, the 3^rd^ centile or above the 97th centile to the expected proportions of 3%.

The worm plot introduced by van Buuren and Fredriks^50^ is a diagnostic tool for checking the residuals for different nonoverlapping ranges of the explanatory variable and can either consist of a collection of detrended Q‐Q plots, each of which applies to a specified age group, or a single worm plot, representing the entire age interval. Residuals are calculated according to age to identify regions or age intervals within which the model fails to fit the data adequately. The shape of the worm indicated how the data differ from the assumed underlying distribution. If the model fits the data well, the worm resembles a flat wormlike string indicating that the data follow the assumed distribution in that age group. Any sudden changes in the shape and location of the worm represent regions where the data has been inadequately modelled. The vertical axis of the worm plot portrays, for each observation, the difference between its location in the theoretical and empirical distributions.

Other types of diagnostics checks though not considered in this paper, as they are not commonly applied in the context of constructing reference centiles include visual predictive checks and normalised prediction distribution errors (NPDE).^51^ Visual predictive checks are simulation‐based diagnostics commonly employed in the field of pharmacometrics to evaluate mixed effects models. Typical summary measures of the distribution are the median and an interval defined by the lower 5% and upper 5% of the values. The NPDE tests for differences from a perfect fit of the model to the data.

##### Application 1: Modelling newborn weight (cross‐sectional design)

**Data**

The data considered here are from the newborns of women who met strict individual eligibility criteria for a population at low risk of fetal growth impairment from the Newborn Cross‐Sectional Study (NCSS) component of the INTERGROWTH‐21^st^ Project.^52^ Anthropometric measurements were taken at birth using an electronic scale (Seca, Germany) to measure birthweight, a specifically designed Harpenden infantometer (Chasmors Ltd, UK) to measure length, and a metallic nonextendable tape (Chasmors Ltd, UK) to measure head circumference (HC).^53^ The NCSS enrolled 59 137 pregnant women at the eight study sites, of whom 20 486 (34.6%) met the individual clinical and demographic eligibility criteria for inclusion in the newborn standards, had a reliable ultrasound estimate of GA, and delivered a live singleton without a congenital malformation. These newborns constituted the NCSS prescriptive subpopulation, and the methods and modelling approaches for cross‐sectional data are illustrated with newborn weight data for boys and girls.

**Statistical modelling**

In this section, multilevel models are not considered because the study is cross‐sectional (newborn weight data for boys and girls) rather than longitudinal. All statistical approaches modelled parameters of the distributions as continuous functions of GA using either FPs or spline methods. Seven candidate models were created with the FP regression method (one two‐parameter model, two three‐parameter models, and four four‐parameter models). One candidate three‐parameter model was created with the LMS method. Two candidate four‐parameter models were created with the LMS extension methods (one LMST and one LMSP). The best model within each class of models was identified, ie, the best two‐, three‐, and four‐parameter models were each identified. Using this information, the single best model across the classes created by a particular approach (either the FP regression method or the LMS method and its extensions) was identified in an add‐up stepwise fashion, starting from the simplest class.

The AIC, BIC, and generalised AIC (GAIC) were used to compare the fit of the models within and across classes.^54^ However, model choice was not based on AIC, BIC, and GAIC alone as other criteria were also considered. For example, how well the fitted models represent the raw data was also considered. In addition, simpler models that showed equally good model fits when compared to more complex models that resulted in small differences in AIC, BIC, or GAIC were preferred. Desirability of having models with the same distribution for the males and females data was also considered. Models that fitted well across the entire GA range were deemed to be better that models with a smaller AIC, BIC, or GAIC but showed inadequate fit or unexpected shifts in centiles, especially at either ends of the distribution.

##### Application 2: Modelling FHC (longitudinal design)

**Data**

The FHC data from the Fetal Growth Longitudinal Study of the INTERGROWTH‐21^st^ Project were obtained prospectively using ultrasound from 14^+0^ weeks until birth (obtained every 5 weeks (± 1 week)) in a cohort of women with optimal health and adequate nutritional status who were at low risk of intrauterine growth restriction. At each visit, FHC was measured three times from three separately obtained ultrasound images in a blinded fashion, ie, previous measurements of each structure were not available to the assessor through suppressed display.^1^ The decision to take triplicate measurements was made at the design stage following advice from an expert ultrasonographer. The study recruited 4233 women who each visited one to six times during pregnancy (95% visited at least four times), giving 20 030 women visits. With three ultrasounds at each visit, 59 973 FHC observations were made across the eight participating sites (117 ultrasound measures were missing).

**Statistical modelling**

Previous studies have shown that the distribution of fetal dimensions is close to normal for any GA^10^ and was the case for FHC data. Therefore, we assumed that, for each GA, FHC had a normal distribution with a mean and SD that varied smoothly with GA.^34^ The best‐fitting powers for the median FHC were obtained by modelling FHC as a function of GA using FPs as previously described. The best FP powers for FHC were provided by an FP2, which takes the general form β_0_ + β_1_*X_1_^p^_1_ + β_2_*X_1_^p^_2_ where powers *p*_1,…,_ *p*_m_ are selected from a restricted set {−2,−1,−0.5,0,0.5,1,2,3} and *x* ^0^ denotes log *(x)* rather than x^0^ = 1. This functional form was then incorporated in a multilevel framework to account for repeated measures.^55^, ^56^ The effect of fitting various multilevel models is evaluated by fitting a two‐level random intercept model, a two‐level random intercept and slope model, and a three‐level random intercept and slope model.

The FHC data had three levels that can be expressed by a simple linear regression model. For a given FHC measurement y_*i*_ (*i* = 1, 2, 3) of subject *j* ( *j* = 1, 2, ..., 4233 subjects) taken on visit *k* (*k* = 1, 2, …,7 visits)

(1) $$y_{\text{ijk}}=\beta_{0+}\beta_{1}{{X_{1}}^{p}}_{1\text{ijk}}+\beta_{2}{{X_{1}}^{p}}_{2\text{ijk}}+\varepsilon_{\text{ijk}.}$$

Equation (1) represents the regression of the FHC, *y*, on the independent variable, GA, in weeks, X. In a typical regression model, the errors, ε_*ijk*_, are assumed to be independent and normally distributed with mean, 0, and variance, σ^2^. However, the independence assumption does not hold for the FHC data set, as it includes repeated measurements of each fetus. Equation (1) assumes that growth across time is the same for all fetuses, ie, that β_0_, β_1_, and β_2_ do not vary by fetus. We therefore included individual‐specific effects to account for data dependency and characterise the difference in growth between individual fetuses. We used three approaches to factor in these fetus‐specific effects and the resulting variation, resulting in three models of increasing complexity.

##### Two‐level random intercept model

In the first multilevel model, the first level of the data was collapsed by taking the average of the triplicate FHC measurements taken at each visit, FHC_1_, FHC_2_, and FHC_3_. The data then comprised two levels, one ultrasound measurement at each visit for each subject during pregnancy (level 1) and all of the measurements from the women at each of the eight sites (level 2). Exploring the influence of each woman on their repeated FHC measurements led to

(2) $$y_{\text{jk}}=\beta_{0+}\beta_{1}{{X_{1}}^{p}}_{1\text{jk}}+\beta_{2}{{X_{1}}^{p}}_{2\text{jk}}+\upsilon_{0j}+\varepsilon_{\text{jk}},$$

where υ_0*j*_ is the influence of individual *j* on their repeat measurements taken on visit *k*. For a given group *j*, the intercept is β_0_ + υ_0j_. The model in Equation (2) is often partitioned into two components in a multilevel framework. The fixed component (within‐subject, level 1) is β_0 +_ β_1_X^p^_1*jk*_ + β_2_X^p^_2*jk*_ and the random component (between‐subject, level 2) is υ_0*j*_ + ε_*ij*_. Equation (2) indicates that a subject's initial FHC measurement at GA (time), *k,* is influenced by that subject's initial level β_0_ + υ_0*j*_ and the population's slope β_1_ and β_2_. Using this relation, each individual has their own initial level. The resulting model is commonly referred to as the random intercept model.^57^ This model was created using Equation (2), averaging the triplicate FHC measurements taken at each visit (N = 20 030).

##### Two‐level random intercept and slope model

The model in Equation (2) uses the same slope, equal to the population slope β_1_ and β_2_, for every fetus. This assumption is too simplistic for our situation, as it is unlikely that every fetus will have the same rate of growth in FHC by GA. Therefore, we relax this assumption and assign each fetus its own initial level (intercept) and slope that vary with GA

(3) $$y_{\text{jk}}=\beta_{0+}\beta_{1}{{X_{1}}^{p}}_{1\text{jk}}+\beta_{2}{{X_{1}}^{p}}_{2\text{jk}}+\upsilon_{0j}+\upsilon_{1j}X_{\text{jk}}+\varepsilon_{\text{jk}}.$$

Equations (2) and (3) model the first level in the same way. Equation (3) includes the term υ_1*j*_, which represents the slope deviation for each subject *j* from the average regression slope β_1_ and β_2_. As before, ε_*jk*_ is an independent error term distributed normally with mean 0 and variance σ^2^.

Using Equation (3), we can obtain two models, ie, by averaging the triplicate FHC measurements taken at each visit or by randomly selecting one of FHC_1_, FHC_2_, and FHC_3_ in each triplicate at each visit. These two models, though different, are based on the same sized data set (N = 20 030).

##### Three‐level random intercept and slope model

So far, we have considered models with only two data levels. The full FHC data set has three levels, ie, triplicate measurements collected at each visit, FHC_1_, FHC_2_, and FHC_3_ (level 1); repeated ultrasound measurements for each woman across multiple visits during the pregnancy (level 2); and measurements taken from many women (level 3). Considering all three data levels, Equation (3) becomes

(4) $$y_{\text{ijk}}=\beta_{0+}\beta_{1}{{X_{1}}^{p}}_{1\text{ijk}}+\beta_{2}{{X_{1}}^{p}}_{2\text{ijk}}+\upsilon_{0\text{jk}}+\upsilon_{1\text{jk}}X_{\text{ijk}}+\varepsilon_{\text{ijk}}.$$

The model developed using Equation (4) considers all levels of data hierarchy (N = 59 973). For all the multilevel models, to assess model fit, the proportion of measurements <3^rd^ or >97^th^ centiles and maximum absolute differences of selected smoothed centiles (ie, 3^rd^, 50^th^, and 97^th^) and empirical centiles were calculated. We discriminated between the models using the respective proportion below the 3^rd^ or above the 97^th^ centiles by comparing the proportions based on the fitted models. The expected proportions were based on the assumption of a normal distribution for FHC at any GA. Model fit was also compared visually using plots of fitted smoothed centiles, Q‐Q plots of residuals against normal scores, and plots of residuals against predicted values.

**Software implementation**

An automated algorithm for selecting FP powers is available in STATA (*xrigls* and *xriml* routines),^58^, ^59^ SAS, and R.^60^ An R package for the LMS method is also available (*lmsqreg*).^61^ The models were fitted using the GAMLSS framework,^62^ available in the statistical software package R.^63^ The GAMLSS model allows the distribution parameters μ, σ, ν, and τ to be modelled as linear, nonlinear, parametric, and nonparametric (smooth) functions of GA. The GAMLSS package provides a comprehensive framework with great flexibility and options for using different methodologies (eg, FP regression and the LMS method), distributions (eg, the skew t‐distribution type 3 and power exponential distribution), smoothing techniques (eg, penalised splines and cubic splines), and diagnostics (eg, worm plots). Multilevel models were fitted using the *runmlwin* package in STATA.^56^ All analyses were performed in STATA version 14 (StataCorp LP, College Station, Texas, USA) and R statistical software version 3.4.2.

## 3 RESULTS

### 3.1 Modelling newborn weight (cross‐sectional design)

**Figure 1:**
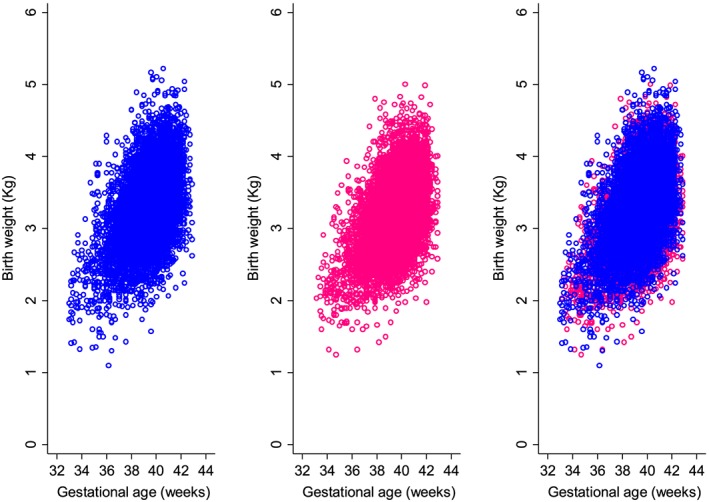
Scatter plot of birthweight measurements according to gestational age for boys (left, blue), girls (middle, pink), and girls and boys superimposed (right) [Colour figure can be viewed at wileyonlinelibrary.com]

**Figure 2:**
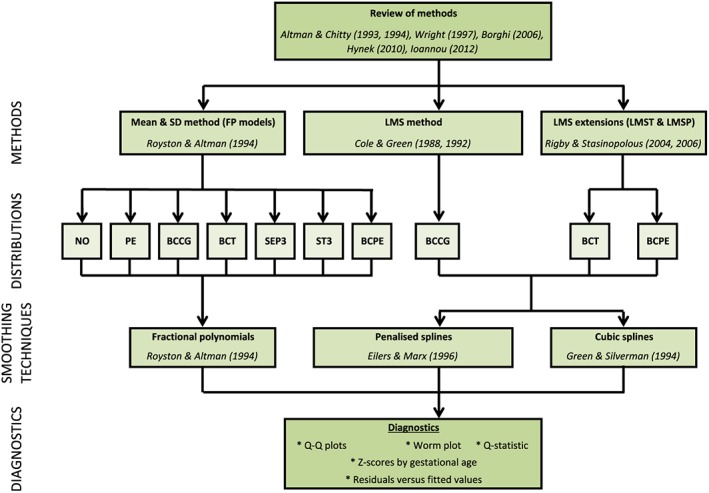
A summary of the most commonly used statistical methodology for analysing growth data. BCCG, Box‐Cox Cole and Green; BCPE, Box‐Cox power exponential distribution; BCT, Box‐Cox t‐distribution; NO, normal distribution; PE, power exponential; SEP3, skew exponential power type 3; ST3, skew t‐distribution type 3; Q‐Q, quantile‐quantile [Colour figure can be viewed at wileyonlinelibrary.com]

**Figure 3:**
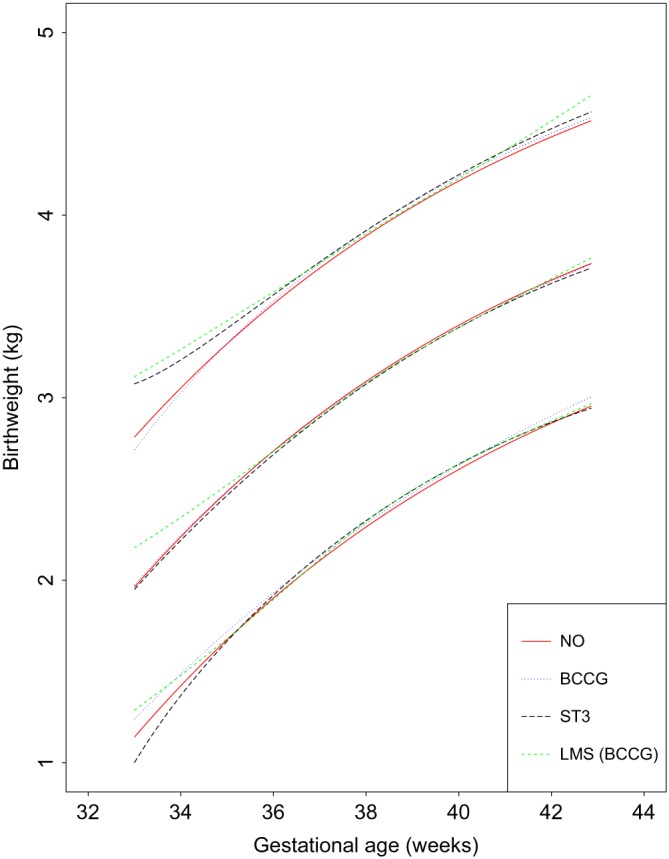
A comparison of fitted centiles for Male birthweight using fractional polynomial regression of selected distributions (ie, normal distribution, Box‐Cox Cole and Green distribution (BCCG), skew exponential power type 3 (ST3)), and the LMS method (LMS (BCCG)) [Colour figure can be viewed at wileyonlinelibrary.com]

Table 1 summarises the number of birthweight measurements according to GA for all of the newborns, divided into boys and girls. Scatter plots of the raw newborn size measurements of birthweight by GA for boys and girls are shown in Figure 1. The distributions of measurements were fairly similar for boys and girls across GA, except at 33 weeks (Table 1 and Figure 1). Figure 2 summarises the statistical methods discussed, associated distributions, smoothing techniques, and diagnostic tests used to evaluate model fit. The newborn data were close to being conditionally normal (“well behaved”) on GA and thus the different methods gave similar results, as Table 2 shows. The decision to model boys and girls data separately was decided a priori due to the expected large sample size to model each precisely.^64^ Table 2 shows the 20 models tested from the four methodological approaches (10 for boys and 10 for girls) and how well each model fitted the newborn weight data. For boys and girls, the lowest AIC, BIC, and global deviance for the FP regression models (M1 to M7) were those based on the skew exponential power type 3 (model M5) and skew t‐distribution type 3 (model M6) distributions (Table 2). Figure 3 compares fitted centiles using FP of selected distributions (ie, normal distribution, Box‐Cox Cole and Green distribution, skew exponential power type 3, and the LMS method (LMS BCCG)).

**Table 1:** Number of birthweight measurements by gestational age for boys and girls

	Birthweight	
	Boys	Girls
Gestational age (completed weeks)	Number of observations	Number of observations
33	34	17
34	48	65
35	128	114
36	323	293
37	857	803
38	2045	1802
39	3009	2869
40	2568	2523
41	1179	1195
42	206	224
**Total**	**10 397**	**9905**

**Table 2:** Summary of birthweight results for the fractional polynomial regression method, LMS, LMST, and LMSP methods. For Bayesian information criterion (BIC), a penalty k = log (n) was applied for the LMS, LMST, and LMSP methods (where n refers to sample size and log is natural logarithm)

		Fractional polynomial regression								LMS method	LMST method	LMSP method
			2 parameters	3 parameters		4 parameters				3‐parameters	4‐parameters	
Anthropometry	Sex	Distribution	normal distribution	PE	BCCG	BCT	SEP3	ST3	BCPE	LMS (BCCG)	LMS (BCT)	LMS (BCPE)
		Model name	M1_B	M2_B	M3_B	M4_B	M5_B	M6_B	M7_B	M8_B	M9_B	M10_B
		Mean	−1, −0.5	−0.5, 0	−2, −2	−2, −2	0.5, 1	0.5, 0.5	−2, −2	mu.df = 3.6	mu.df = 5.8	mu.df = 6.0
	Male	SD	−2	−2	−2	−2	3	3	−2	sigma.df = 3.3	sigma.df = 7.5	sigma.df = 7.4
		Nu	NA	3	−2	−2	−2	−2	−2	nu.df = 2.0	nu.df = 2.0	nu.df = 2.0
		Tau	NA	NA	NA	−2	−2	−2	3	NA	tau.df = 2.0	tau.df = 2.0
	Goodness of fit	% below 3^rd^	2.67	2.39	3.08	3.04	2.97	3.09	2.92	3.05	3.02	2.90
		centile										
		% above 97^th^	96.49	96.59	96.86	96.97	97.15	97.02	97.08	96.76	96.88	96.99
		centile										
		Global deviance	531	476	500	432	434	428	445	477	419	427
		‐11 000										
		AIC‐11 000	547	498	522	460	456	452	473	495	438	446
		BIC‐11 000	641	578	602	562	545	539	574	559	506	514
Birthweight	Female	Distribution	normal distribution	PE	BCCG	BCT	SEP3	ST3	BCPE	LMS (BCCG)	LMS (BCT)	LMS (BCPE)
		Model name	M1_G	M2_G	M3_G	M4_G	M5_G	M6_G	M7_G	M8_G	M9_G	M10_G
		Mean	3, 3	3, 3	3, 3	3, 3	3, 3	3, 3	3, 3	mu.df = 3.5	mu.df = 5.5	Mu.df = 5.6
		SD	3	3	−2	−2	3	3	−2	sigma.df = 3.0	Sigma.df = 4.0	Sigma.df = 4.3
		Nu	NA	3	3	3	−2	−2	3	nu.df = 2.0	nu.df = 2.0	nu.df = 2.0
		Tau	NA	NA	NA	3	0.5	3	3	NA	tau.df = 2.0	tau.df = 2.0
	Goodness of fit	% below 3^rd^	2.36	2.18	2.96	2.98	2.87	2.97	2.83	2.96	3.02	2.82
		centile										
		% above 97^th^	96.33	96.44	96.82	96.95	97.00	96.90	97.00	96.85	96.94	97.01
		centile										
		Global deviance	168	107	106	49	52	50	50	98	33	40
		‐10 000										
		AIC‐10 000	184	129	128	77	76	74	78	115	60	61
		BIC‐10 000	241	208	207	177	62	161	179	175	158	135

Abbreviations: AIC, Akaike information criterion; BCCG, Box‐Cox Cole and Green; BCPE, Box‐Cox power exponential distribution; BCT, Box‐Cox t‐distribution; df, degrees of freedom; NO, normal distribution; PE, power exponential; SEP3, skew exponential power type 3; ST3, skew t‐distribution type 3.

**Figure 4:**
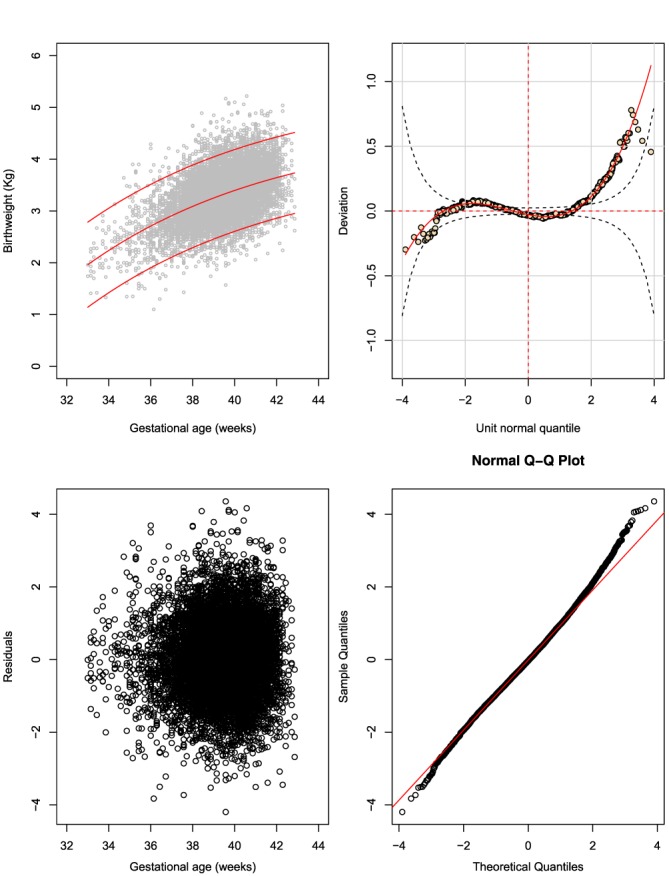
The fractional polynomial regression method: fractional polynomial fit of a two‐parameter model assuming a normal distribution (two powers for the mean and one for the SD) for male birthweight (Model: M1_B, Table 2). The plot shows (A) the fitted 3^rd^, 50^th^, and 97^th^ smoothed centiles according to gestational age (top left panel), (B) a worm plot (top right panel), (C) a scatter plot of the residuals according to gestational age (bottom left panel), and (D) normal quantile‐quantile (Q‐Q) plots of the distribution of Z‐scores (bottom right panel) [Colour figure can be viewed at wileyonlinelibrary.com]

We use Figure 4 for male birthweight (fractional polynomial regression method) as a representative example. The top left panel shows the fitted 3^rd^, 50^th^, and 97^th^ smoothed centiles according to GA. It provides a visual assessment of whether the smooth centiles offer a good representation of the raw data overall and at both ends of GA. In this plot, one can see a tendency for slight deviations of model fit for the 97^th^ centile to areas with no data at GA <35 weeks, ie, some overestimation. The top right panel shows a worm plot. The red curve in the plot is a penalised spline polynomial fitted to the points on the plot. In this plot, the worm plot is flat for most of the middle age range, but changes shape and deviates from the expected zero line at lower and upper GAs. This is a clear indication of inadequate model fit for male birthweight data. The bottom left plot shows a distribution of residuals as a function of GA. The bottom right plot shows a normal Q‐Q plot of Z‐scores. It evaluates whether the residuals have a close‐to‐normal distribution represented by a straight diagonal line cutting through the plot. In this plot, similar to deductions made from the worm plot, there is deviation from a normal distribution at the bottom and top ends of the distribution, representing the lower and upper ranges of GA. However, the number of observations away from the line are a small percentage of the total sample.

**Figure 5:**
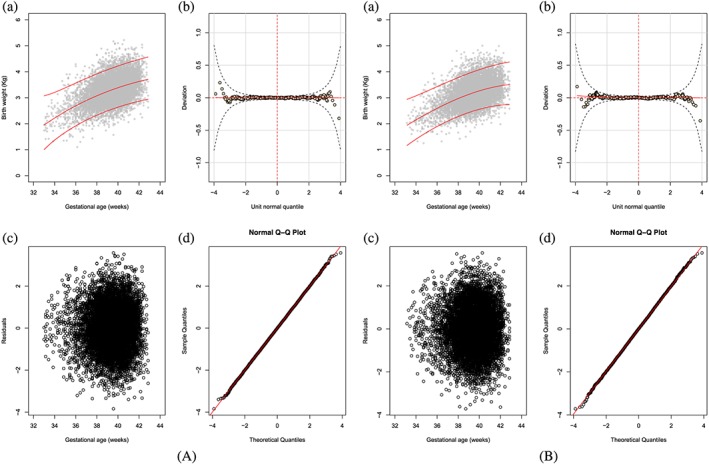
Panel A is MALES and panel B is FEMALES the fractional polynomial regression method: fractional polynomial fit of a four‐parameter model assuming a skew t‐distribution type 3 distribution (two powers for the mean, one for the SD, one for skewness, and one for kurtosis) for male birthweight (Model: M6_B, Table 2). The plot shows (a) the fitted 3^rd^, 50^th^, and 97^th^ smoothed centiles according to gestational age (top left panel), (b) a worm plot (top right panel), (c) a scatter plot of the residuals according to gestational age (bottom left panel), and (d) normal quantile‐quantile (Q‐Q) plots of the distribution of Z‐scores (bottom right panel) [Colour figure can be viewed at wileyonlinelibrary.com]

The skew t‐distribution type 3 distribution provided the best fit for boys and girls, judging by the fitted smoothed centiles, which offer a good representation of the data; the completely flat worm plots; and the Q‐Q plots following a straight line on the expected line (Figures 5A and 5B).

### 3.2 Modelling FHC (longitudinal design)

**Figure 6:**
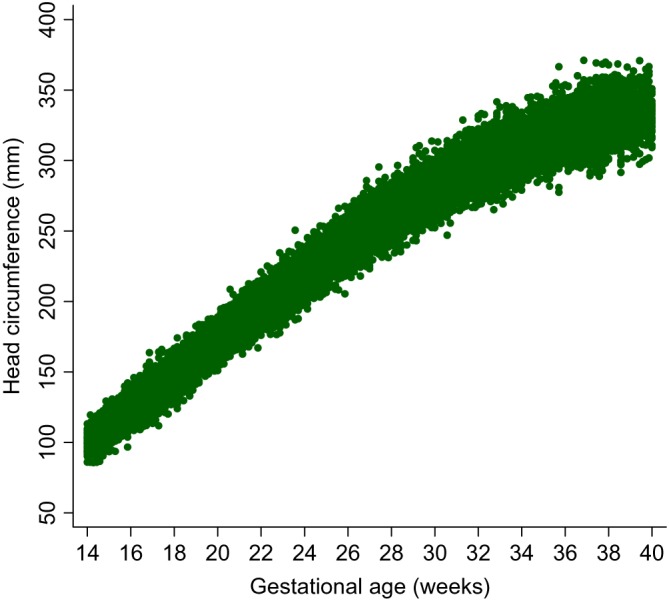
Scatter plots of the raw fetal head circumference measurements by gestational age for all of the sites combined [Colour figure can be viewed at wileyonlinelibrary.com]

**Figure 7:**
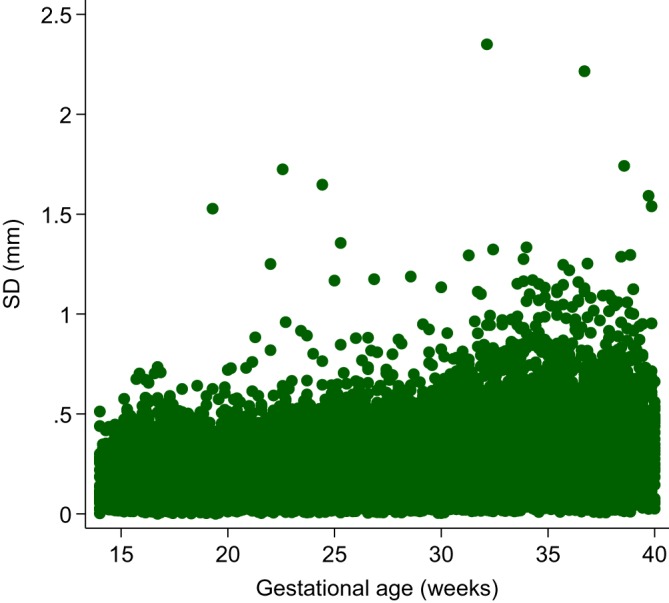
Variance of each set of triplicate measurements at each visit for all women according to gestational age [Colour figure can be viewed at wileyonlinelibrary.com]

Table 3 shows a descriptive summary of the number of women and the number of follow‐up visits each woman attended during pregnancy. Most of the women (93%) attended at least four follow‐up visits during pregnancy. Figure 6 shows a scatterplot of the raw FHC data by GA collected from all women from all eight sites. Figure 7 shows the variance of each set of triplicate measurements at each visit for all women according to gestational age.

**Table 3:** Summary of the number of women at each visit and the total number of follow‐up visits at which fetal head circumference was measured

**Number of**	**Number of women**	**Percentage**	**Number of women**	**Percentage**
**follow up visits (X)**	**who visited only**		**who visited at least**	
	**X times**		**X times**	
1	39	0.9	4233	100.00
2	55	1.3	4194	99.1
3	203	4.8	4139	97.8
4	810	19.1	3936	93.0
5	2724	64.4	3126	73.8
6	402	9.5	402	9.5
**Total**	**4233**	**100.00**	**20 030**	**100.00**

**Figure 8:**
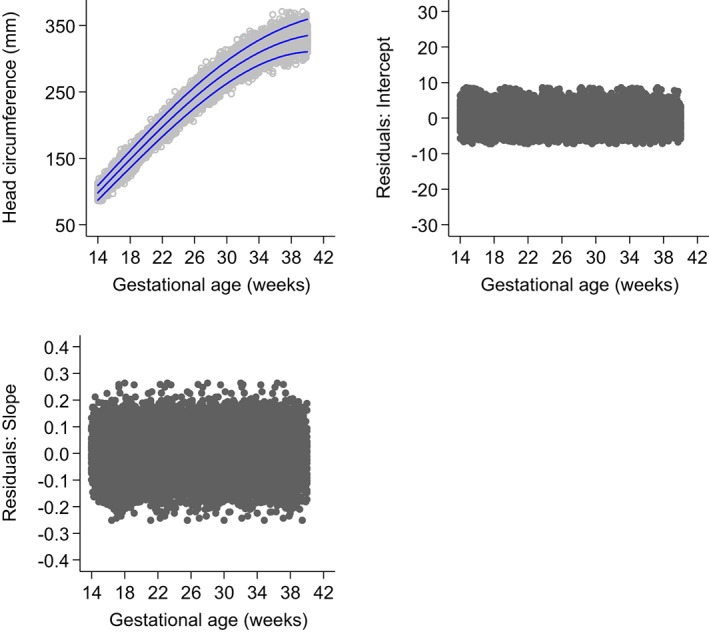
Fitted 3^rd^, 50^th^, and 97^th^ smoothed centile curves (dashed blue lines) for fetal head circumference (mm) by ultrasound according to gestational age (weeks), showing the actual observations (open grey circles) (top left), plot of intercept residuals against gestational age (top right), and slope residuals against gestational age (bottom left), of a three‐level random intercept and slope multilevel model applied to all fetal head circumference triplicate measurements taken at each visit [Colour figure can be viewed at wileyonlinelibrary.com]

Table 4 contains the specifications and goodness of fit of the four multilevel models fitted to the FHC data. An FP with two powers for the mean was first fit for FHC data, and then modelled within a multilevel framework to account for repeated measures. The four multilevel models, though formulated differently, had reasonably similar results. Figure 8 shows the fitted 3^rd^, 50^th^, and 97^th^ smoothed centiles across GA of a three‐level random intercept and slope multilevel model applied to all FHC triplicate measurements taken at each visit, intercept, and slope residuals according to gestational age.

**Table 4:** Model details and results of the multilevel modelling

			Fractional			Goodness of fit	
			polynomial powers				
Model specification	Detail	N	Median	SD	Deviance	Observations	Observations
						<3^rd^ centile	>97^th^ centile
Random intercept	Take the mean of the	20 030	2, 2	1	136 690	638 (3.2%)	647 (3.2%)
two‐level model	triplicate FHC						
	measurements						
	for each visit						
Random intercept	Take the mean of the	20 030	2, 2	1	132 845	866 (4.3%)	923 (4.6%)
and slope	triplicate FHC						
two‐level model	measurements						
	for each visit						
Random intercept	Randomly select	20 030	2, 2	1	135 710	926 (4.6%)	1009 (5.0%)
slope and	one of the three FHC						
two‐level model	measurements						
	for each visit						
Random intercept	Consider all	59 973	2, 2	3, 3, 3	346 036	2993 (5.0%)	3231(5.4%)
and slope	three data levels						
three‐level model							

The random intercept model differed by at most 3.4 mm and 3.8 mm at the 3^rd^ centile from the random intercept and slope two‐level models. The two two‐level random intercept and slope models were very similar, with a maximum absolute difference of <0.5 mm at the extreme centiles. The two‐level random intercept model differed by 4.7 mm at the 3^rd^ centile from the three‐level random intercept and slope model and by 2.4 mm at the 97^th^ centiles from the two‐level random intercept and slope model based on randomly selecting one FHC measurement from each triplicate. However, it is worth noting that the models all differ in terms of precision.

The final model chosen for FHC was the three‐level random intercept and slope model with an FP2 model for the mean FHC and an third‐order FP for the SD^1^ (Figure 8, Table 4). The resulting equations for FHC was

**Table:** 

Fetal head Circumference (FHC)	Mean	−28.2849 + 1.69267*GA^2^–0.397485*GA^2^ *log (GA)
	SD	1.98735 + 0.0136772*GA^3^–0.00726264*GA^3^*log (GA) + 0.000976253*GA^3^*log (GA) ^2^
All log are natural logarithms. GA = exact gestational age in weeks.		

## 4 DISCUSSION

We have described the principal methodologies available for the construction of age‐specific reference or standard centiles using cross‐sectional and longitudinal data. We have demonstrated their application using the recently published INTERGROWTH‐21^st^ newborn data for weight^52^ and FHC.^3^ The choice of methodology is important as inaccurate centiles resulting from inferior methods can lead to incorrect judgements about fetal size development, resulting in suboptimal clinical care.^37^

Choosing the best model from amongst many is not trivial, especially when dealing with large data sets such as the INTERGROWTH‐21^st^ data. Significance testing and goodness‐of‐fit statistics like the likelihood ratio test, BIC, or the AIC are usually used to discriminate between models. However, these methods tend not to be useful when examining large data sets, as very small differences are statistically significant even though the models are indistinguishable on actual centile plots. Reference or standard centiles should ideally be produced that have the best fit to the data and change smoothly with GA, using as simple a statistical model as possible that can easily be transformed into Z‐scores (SDS scores), to ensure comparability and usability. The expected distribution of SDS is normally distributed with μ = 0, and σ = 1. The mean, μ = 0, represents the expected average growth. Similarly, values outside ±2SDS are usually indicative of excessive growth (≥ +2SDS) or inadequate growth (≤ ‐2SDS). These values are usually indicative of growth restriction or other clinical complications affecting growth hence warranting further investigations.

Model choice should not be based on statistical considerations alone, however, but also on the quality of the fit to the data, ie, model fit across the GA range. For cross‐sectional data, we explored a variety of methods and models for fitting reference or standard centiles based on the modelling framework that was used in the INTERGROWTH‐21^st^ Project. Royston and Altman demonstrated the utility and flexibility of FPs for modelling growth data that is typically nonlinear.^40^ Fractional polynomial regression wase used to obtain the respective FP powers relating FHC as a function of GA by modelling the mean and SD. These powers were then incorporated in a GAMLSS framework to model skewness and kurtosis. The values for skewness and kurtosis were constant but nonzero, as they did not vary with GA. In selecting the best model, model fit was evaluated both visually and formally using statistical tests.^10^, ^15^, ^64^ Considerations such as identifying a common distribution for boys and girls that best represents the birthweight data were taken into account. We preferred to use the same distribution for boys and girls, even though their data were modelled separately.

Based on these considerations, a skew t‐distribution (type 3)^65^ with four parameters (μ, σ, υ, and τ) was selected as the most appropriate distribution for constructing birthweight curves for boys and girls. We had not anticipated that modelling the birthweight data would need a more complex distribution than the normal distribution. We believe the requirement for more complex distributions can be explained by our carefully selected population of healthy women. These women primarily had good pregnancy outcomes. This led to what is commonly referred to as “data heaping,” ie, very few deliveries were observed in early gestation (<34 weeks) as most of the women had term deliveries (≥37 weeks). This data heaping posed challenges for the data modelling due to the nonuniform distribution of the data across GA. Having significantly more data points in late gestation affected the fit at the bottom end of the distribution. A more complex distribution that accounted for skewness and kurtosis was therefore required. Data heaping can also be overcome by selecting a subsample of observations to artificially construct a database with a balanced number of observations over the range of GA or weight measurements. However, this method discards and wastes data, and is not recommended given the time and cost associated with obtaining the data.^66^

The LMS method is widely used for modelling anthropometric data due to its flexibility, ability to account for skewness, and closed formulation to the normal distribution based on the L, M, and S curves. The LMS method provided excellent summary statistics though it did not perform well with the sparse data near the end of the age range (due to edge effects)^11^ as depicted by the diverging worm plot beyond ±3SD. However, it is worth noting that ±3SD corresponds to the 0.1th and 99.9th centiles, which are far more extreme centiles than the 3^rd^ and 97^th^, and therefore, judgements on overall model fit should be interpreted with caution.

Similarly, for longitudinal data, we used multilevel models that account for subject‐specific variations in growth by allowing subject‐specific random effects.^67^ Other multilevel modelling approaches that have been applied in a similar context include the brokenstick model by van Buuren,^68^ and linear spline models discussed in the work of Howe et al.^69^ We identified modelling approaches that offered a good fit to the raw data and accounted for increasing variability with GA, which is a phenomenon observed in growth data. We analysed FHC data and modelled the data using multilevel models with increasing levels of complexity that accounted for the data hierarchy in different ways. One limitation observed with the multilevel approach is the tendency for bias as evaluated by the number of observations <3^rd^ or > 97^th^ centiles compared to the expected proportion of 3%. The degree of bias increased with complexity of the mixed effects model. Villamor and Bosch^70^ and Cole and Cortina Borja^71^ have also demonstrated through simulation studies that the median of three replicates is a robust approach, even in the presence of high levels of contamination, to combine participants' raw data values for use in analyses as opposed to the mean. We only considered the mean of the triplicate measurements and therefore acknowledge this is a possible limitation of our analyses.

In summary, during study planning, the overall goal of the study must be determined and an appropriate study design must be chosen to answer the specific questions or hypotheses that avoids either wasted effort or later data wastage. For example, if a study aims to develop references or standards, a well‐designed cross‐sectional study with data collected specifically for this purpose is sufficient. Model choice is dependent on the study aim and the question one is trying to answer rather than the richness of the data that could be collected.

We have described the FP regression, LMS, LMST, and LMSP methods and illustrated them using birthweight data from the INTERGROWTH‐21^st^ Project. The methodology and statistical considerations discussed here were also applied to the newborn birth length and birth HC data. These considerations and methodology were key to developing the international newborn standards.^52^ We have also demonstrated modelling repeated measures data using FHC data collected in the Fetal Growth Longitudinal Study component of the INTERGROWTH‐21^st^ Project. These methods are not restricted to fetal data and can be applied to other repeated measures data. The methodology and statistical considerations discussed here were also applied to other commonly measured fetal dimensions, such as biparietal diameter, occipito‐frontal diameter, abdominal circumference, and femur length.

## COMPETING INTERESTS

The authors declare that they have no competing interests.

## AUTHORS' CONTRIBUTIONS

Eric O. Ohuma performed the statistical analysis and wrote the manuscript. Douglas G. Altman read initial versions of the manuscript.

## AUTHORS' INFORMATION

Eric O. Ohuma is a Senior Medical Statistician and Douglas G. Altman is Professor of Statistics in Medicine.
